# Social determinants of health that permeate adolescent students’ mental distress

**DOI:** 10.1590/0034-7167-2025-0281

**Published:** 2026-07-17

**Authors:** Luiza Maria Rabelo Silva, Sabrina Silva Martins, Larissa de Almeida Rezio, Pâmela Roberta Oliveira, Marina Nolli Bittencourt

**Affiliations:** I Universidade Federal de Mato Grosso. Cuiabá, Mato Grosso, Brazil; II Universidade Federal de Mato Grosso. Barra do Garças, Mato Grosso, Brazil

**Keywords:** Social Determinants of Health, Adolescent, Mental Health, School Health Services, Psychological Distress., Determinantes Sociales de la Salud, Adolescente, Salud Mental, Servicios de Salud Escolar, Distrés Psicológico.

## Abstract

**Objectives:**

to analyze the social determinants of health involved in adolescent students’ mental distress in the municipality of Cuiabá.

**Methods:**

a qualitative study, linked to a matrix project, based on the social determinants of health theoretical model by Solar and Irwin. Data came from a matrix research study, analyzing 98 interviews with adolescents aged 12 to 18 years. The interviews were guided by a self-assessment instrument for emotional aspects and social competencies: Youth Self-Report. Qualitative data analysis was carried out using the thematic analysis proposed by Braun and Clarke.

**Results:**

in light of the adopted theoretical model, three subcategories emerged from the structural determinants of health category, and six subcategories from the intermediate determinants of health category.

**Final Considerations:**

by using the Solar and Irwin model, it was possible to identify that the social determinants of health play an important role in manifestation of mental health symptoms among adolescents.

## RESUMEN

**Objetivos:**

analizar los determinantes sociales de la salud relacionados con el sufrimiento mental de adolescentes en edad escolar del municipio de Cuiabá.

**Métodos:**

estudio cualitativo, vinculado a un proyecto matricial, basado en el modelo teórico de determinantes sociales de la salud de Solar e Irwin. Los datos procedían de una encuesta matricial que analizó 98 entrevistas con adolescentes de entre 12 y 18 años. Las entrevistas se guiaron por un instrumento de autoevaluación de aspectos emocionales y habilidades sociales: *Youth Self-Report*. El análisis cualitativo de los datos se realizó mediante el análisis temático propuesto por Braun y Clarke.

**Resultados:**

a la luz del modelo teórico adoptado, surgieron tres subcategorías de la categoría de determinantes estructurales de la salud y seis subcategorías de la categoría de determinantes intermedios de la salud.

**Consideraciones Finales:**

mediante el modelo de Solar e Irwin, se identificó que los determinantes sociales de la salud desempeñan un papel importante en la manifestación de síntomas de salud mental en adolescentes.

## INTRODUCTION

The social determinants of health (SDH) approach broadens the understanding of the health-disease process by interconnecting knowledge from multiple areas, such as public policies, social, economic, and cultural aspects. This perspective considers living conditions throughout the entire life cycle, shaping the incidence of diseases and risks to public health^(1)^. In this regard, the World Health Organization (WHO) created a commission dedicated to investigating the effects of social SDH in the early stages of life, highlighting factors that affect child development in family, school, community, and socio-political contexts^(2)^.

Research shows that disruptions in contexts associated with SDH can destabilize individuals’ lived experiences, increasing their vulnerability to infections, mental disorders, eating disorders, drug use, self-harm, early sexual initiation, and adolescence pregnancy^(3)^.

In the last decade, the growth of psychiatric disorders has repositioned mental illness as a global social and public health issue. Beyond being a fundamental right, mental health is a pillar for individual and socioeconomic progress within communities^(4)^.

Therefore, analyzing the repercussions of SDH on mental health at different stages of life is essential, as susceptibility to these factors varies according to individual trajectories^(5)^. Furthermore, it is noteworthy that events occurring during critical periods of development, such as early childhood, have more severe effects on triggering mental health problems^(6)^.

In this regard, Prokosck *et al*.^(7)^ identified that stressful situations and traumas in childhood harmed young adults’ mental health, triggering anxiety and disruptive behaviors. During the pandemic, adolescents in Kenya and Nigeria^(8)^ faced worsening social conditions and new challenges, such as hunger, school closures, and social isolation, which intensified anxiety, early pregnancies, and unions, as well as episodes of physical, sexual, and emotional abuse^(8)^. Similarly, in Oiapoque, in the far north of Brazil, it was found that determinants such as abandonment, violence, bullying, hunger, and child abuse were directly associated with psychological distress in children^(3)^.

Such evidence reinforces the need for public policies that promote mental health and offer psychosocial support to children and adolescents, through the articulation between education, health, and social assistance. In response to this demand, Law 14,819, enacted in January 2024, established the Brazilian National Policy for Psychosocial Care in School Communities, promoting integrated actions between schools, the School Health Program, the Unified Social Assistance System, and psychosocial care networks^(9)^.

Synergy between sectors and prioritizing school settings are key strategies, since the school is a central space for cognitive and emotional development, in addition to allowing the tracking of risks and protective factors.

Given the gaps and challenges highlighted in the literature, such as inadequate professional training, lack of integration between support networks, and a lack of clear policy guidelines, it becomes essential to understand SDH that influence adolescents’ mental health in order to support more effective interventions. Hence, the research seeks to answer the following question: how are mental SDH expressed among adolescent students in Cuiabá in light of SDH model developed by Solar and Irwin?

## OBJECTIVES

To analyze SDH involved in adolescent students’ mental distress in the municipality of Cuiabá.

## METHODS

### Ethical aspects

This study is part of a matrix project previously authorized by the State Department of Education and approved by the *Universidade Federal do Mato Grosso* Research Ethics Committee. Since it uses secondary data, it did not require further ethical authorization, maintaining the principles of confidentiality, anonymity, and non-maleficence, in accordance with Resolution 466/2012. The research respected the right to information, privacy, voluntary participation, and withdrawal of consent, without prejudice or risk to participants’ health.

### Theoretical-methodological framework

SDH reflect a broader view of the health-disease process^(10)^, and, according to Buss and Pellegrini Filho^(11)^, refer to the conditions in which people “are born, grow, live, work and age”, influenced by social, economic, behavioral, cultural, ethnic/racial, and psychological factors that directly affect health and quality of life.

Currently, the WHO adopts the Solar and Irwin model, presented at the World Conference on Social Determinants of Health in 2011^(12)^, which distinguishes between structural and intermediate determinants. While structural determinants include factors such as public policies, governance systems, the labor market, education, and social values that shape socioeconomic stratification^(13)^, intermediate determinants involve material living and working conditions, psychosocial and behavioral factors, and the health system. It is noteworthy that the latter is considered an intermediate determinant due to barriers to access, and that social cohesion and capital cut across the dimensions of the model^(13)^.

Finally, the Brazilian National Commission on Social Determinants of Health^(12)^ highlights structural determinants as central to understanding health inequities, resulting from power asymmetries and the socioeconomic and political context. In the case of mental distress among school adolescents, this model shows how poverty, violence, family instability, and school settings negatively impact mental health. Support networks and access to mental healthcare services, on the other hand, can reduce these effects, highlighting the importance of intersectoral actions to promote a welcoming and equitable school settings.

### Study design

This is a qualitative, descriptive, and exploratory study, using Solar and Irwin’s SDH model as a theoretical framework^(13)^.

### Methodological procedures

#### Study setting

The study took place in seven regular state high schools located in urban areas of the capital of Mato Grosso, three of which operated on a full-time schedule. These schools were selected for convenience.

#### Data source

The data were collected from a database containing 222 interviews with adolescents aged 12 to 18, an age range defined based on the Statute of the Child and Adolescent^(14)^, and on the Youth Self-Report (YSR) theoretical foundations^(15)^, which was the instrument that guided the interviews. Interviews with adolescents who presented responses indicative of mental distress according to the YSR and who related them to experiences that allowed the symptoms to be correlated with SDH were included in this research. Incomplete interviews and those with low-quality audio were excluded.

#### Data collection and organization

Data collection for the school interviews occurred in two stages. The first stage took place between March 2021 and December 2022 in seven state schools in the capital of Mato Grosso, with a team composed of nursing students, a professor, and a doctoral candidate. After selecting the schools and obtaining authorization from the principals, the researchers presented the study to professors and students, distributing the Informed Consent Form (ICF) to parents/guardians. Students with signed ICF and Informed Assent Forms participated in data collection.

The interviews and the application of the instruments took place in private rooms, lasting an average of 45 minutes. The data were collected digitally via Google Forms^®^, using YSR. The interviews were recorded, and relevant accounts were explored in greater depth with the open-ended question “Tell me more about it”.

The second stage began after the audio recordings were transcribed. Of the 222 interviews conducted, 98 adolescents presented psychopathological symptoms, and 32 interviews were selected for analysis because they showed relationships between mental distress and SDH, according to the Solar and Irwin model^(13)^.

#### Data analysis

Data were examined using thematic analysis by Braun and Clarke^(16)^, in which, in phases one to four, SDH model of Solar and Irwin^(13)^ was used as a theoretical basis. The application of this methodology unfolded in six phases, and the analysis revealed two main categories. Category 1, referring to the structural determinants of health inequalities, encompasses three subcategories: Recurring thoughts of worry about the future; Influence of interpersonal relationships on the recurrence of thoughts; and Low social protection as a trigger for mental distress. Category 2, corresponding to the intermediate determinants of health, includes six subcategories: Aggressive behavior and anger as a consequence of lived experiences; Experiencing bullying and its repercussions on mental distress; Impact of self-image on mental health; Psychosocial factors and non-suicidal self-harm; Psychosomatic complaints arising from distress; and Mental distress related to family health.

## RESULTS

### 1 - Structural determinants of health inequalities

This category encompasses socioeconomic status, public policies, culture, and values.

#### 1.1 Recurring thoughts of worry about the future

Adolescents expressed anxiety about the future, revealing concerns about employment, studies, and family responsibilities:

*Worrying about finding a job and helping my parents, taking a course, and who knows what else.* (P99)

*Concern about the future* [...] *about college, about studying, about work.* (P103)

P6 revealed excessive concern with the caregiver role in the family, indicating premature responsibility, even at the expense of their own needs:

[...] *how am I supposed to live with my family, considering I’m basically the caretaker of the house and I have to stay there to help them* [...] *so, I just want to make them proud* (P6).

Similarly, P15 revealed a constant demand for performance in areas such as studies, work, and personal care, while P118 demonstrated a self-perception marked by high expectations regarding academic performance:

[...] *I keep thinking like this all the time, “Oh, I need to make this work”. Like, this year there’s the ENEM exam; I want to pass* [...] *I need to dedicate myself, I need to give my best in this. Or, if I’m at work, I’m like, “Hang in there, don’t freak out”* [...] *at the gym, I’m like, “Oh, come on, you can do it”. It’s always like that.* (P15)

[...] *I think a lot about school, I think I think too much about my grades. I think I still put too much pressure on myself about it.* (P118)

In addition to self-criticism, P15’s and P118’s reports indicated an attempt at emotional repression, as in the expressions “hang in there” and “do not freak out”.

#### 1.2 Influence of interpersonal relationships on the recurrence of thoughts and self-perception

Interpersonal relationships influence the recurring thoughts of adolescents, with family conflicts, insecurity in friendships, and academic performance identified as the main triggering factors, as per the following response:

*Worries about my family, about my friends.* (P139)

Insecurity in interactions appeared in responses such as:

*If I send a message to someone, and they don’t reply or reply in a way I didn’t expect, I wonder if I sent the right message or if I made the right choice.* (P100)

*I find myself comparing myself to others a lot, and sometimes I feel like people are leaving me out* [...] *I have a friend I tell everything to. Then we have another friend, and she kind of leaves me out. I feel really bad about it.* (P125)

Social comparisons emerged as a significant factor, as shown in P125’s report, revealing feelings of low self-esteem, insecurity, or not feeling loved. Furthermore, academic performance is also a constant concern, associated with physical symptoms of anxiety and cognitive difficulties, as reported by P142:

*Like when I get a bad grade. That’s when I get worried. I mean, even before the exam, I get worried.* [...] *it gives me anxiety, I start sweating.* (P142)

Furthermore, constant self-reflection on past mistakes emerged in several statements, such as those of P122 and P153:

*Sometimes I do something wrong, and then I spend, like, a week thinking about it.* (P122)

*Like, if I mess something up, I keep thinking about it.* (P153)

It was also highlighted that the need for external validation impacts self-image and self-esteem, as evidenced by P149:

[...] *if someone compliments me, I’ll keep dwelling on it until I feel the same way. And if someone says I’m good at something, I’ll keep dwelling on it until I’m convinced and no longer think I’m good at it.* (P149)

#### 1.3 Low social protection as a trigger for mental distress

The school emerged as a space of insecurity, generating fear and stress:

[...] *I’m worried that someone might come and commit a massacre here at the school. Because that day happened at IFMT. A boy locked all the students in a room* [...] *he attacked the students in the same classroom and the professor, and tried to stab them.* (P101)

*I’m scared because someone’s already been stabbed here. I don’t know if it was with a knife or a pen, it happened right there in that room, I think it was in the school classroom. Room twelve, this one here, and, man, it’s covered in blood. They showed me the videos, the video, and the girl has already been arrested.* (P157)

The feeling of insecurity also extends to other social spaces, such as the streets, where adolescents experienced situations of harassment and abuse, with the fear of gender-based violence being evident in P139’s report:

*I’m afraid someone will abuse me on the street. Yeah, I’m really scared that I’ll be walking, and then someone will come up and grab me. And also harassment. I’m very scared* [...] *one day, I was coming to school with my mother* [...] *a black car passed by me and my mother was further ahead, when I was going slowly* [...] *he said, “Hey, little kitty”, and I got scared, I started to cry.* (P139)

Furthermore, the poor condition of public roads also contributes to insecurity:

*I’m afraid of holes. Manholes, more specifically. That I fell into a sewer. I was looking at the moon.* [...] *it was a hole that went all the way around my leg. I needed 54 stitches.* (P30)

### 2 - Intermediate determinants of health

This category addresses behavioral, psychosocial factors, and material circumstances.

#### 2.1 Aggressive behavior and anger as a consequence of lived experiences

Adolescents reported that negative interactions generated irritation, anger, and aggressive thoughts associated with conflicting family dynamics, rejection, and lack of emotional support.

An adolescent exemplified the frustration in the face of unfair treatment:

[...] *for some reason, I get quite annoyed when they treat me badly and with ignorance.* (P140)

Another adolescent expressed fear of their own anger and the aggressive thoughts it can generate:

[...] *I’m afraid of picking up a knife and stabbing someone. I’m afraid of pushing someone in front of a car. I’m afraid of going into my mother’s room and hitting her in anger. That would be an outburst, because I’m also very angry about getting up in that church and hitting a child in the face, because a child who makes a lot of noise deserves a good beating, because I have a certain trauma because of that.* (P149)

#### 2.2 Experiencing bullying and its repercussions on mental distress

The accounts indicated that school bullying exacerbates negative feelings, but family support helped to give new meaning to these experiences.

*They saw me, looked me in the eye, and made that vomiting sound. How awful.* [...] *I was bullied a lot at my old school. Then my stepmother turned around and told me not to worry about it, because I was a very special girl and their opinion didn’t matter. So, I kept repeating that in my head* [...] *there are a lot of people who don’t like me, like, 95% of the school.* (P52)

Bullying also generates body insecurity, as reported by P154:

[...] *people keep making fun of me and I don’t like it.* [...] *I am very insecure about my body.* (P154)

One report also indicated that bullying can, in some cases, lead to suicidal thoughts:

*I’ve already tried* [...] *there was one thing that hurt me so much that I really wanted to: it was bullying. I suffered a lot of bullying when I was little* [...] *I was bullied at school by other children because I was overweight.* (P7)

#### 2.3 Impact of self-image on mental health

It also became evident that dissatisfaction with one’s appearance generates distress and low self-esteem, as demonstrated by the following account:

[...] *I think I’m very ugly.* [...] *it seems like there are clothes that just don’t look right on me. Like, I think I’m too fat* [...] *so, it seems like when I wear pants, I look ugly, because there are other girls who look good.* (P106)

Furthermore, it generates the desire to have a different body, as reported:

*I wanted to be very thin* [...] *I want to weigh 48 kilos; I’m trying to reach that goal.* [...] *I wish I had it, but I don’t have anorexia nervosa.* (P24)

#### 2.4 Psychosocial factors and non-suicidal self-harm

The interviews revealed that rejection, family conflicts, and the influence of friendships are associated with self-harm. In the following statement, the feeling of rejection and judgment by others manifested itself as a factor in psychological distress:

[...] *because I feel like people don’t like me, that everyone is judging me, that everyone is giving me dirty looks. Because, basically, they don’t understand what I’m feeling.* (P154)

Furthermore, social exclusion by the family, as a triggering factor for self-harm, was evidenced in the following response:

[...] *and what makes me have these thoughts and do these things* (self-harm) *is my parents, because sometimes I feel like my brother is, like, their favorite, and they prefer my brother more than me, you know?* [...] *and then I feel very sad. I feel excluded* (P132)

According to reports, family conflicts are also triggers for non-suicidal self-harm:

[...] *it happened like this: I was at home and my mother came to talk to me. I don’t know what happened, but I got angry with her, and I said something that made her cry. I cried all night. I regretted it a lot, so much so that I even cut myself one day because of what happened.* (P157)

[...] *I really, really, really want to take very high doses of medication just to travel, because my reality with my mother is very difficult.* (P24)

Furthermore, there are reports of the influence of friendships on this behavior:

[...] *sometimes I would fight with my parents, and these online friends I have, I had a friend* [...] *we met on Instagram^®^ and we talked a lot, but* [...] *we had a fight and I just got really sad and I cut myself.* (P132)

*I’ve cut myself before, but only because of other people. But because I saw a friend doing it, she said it was good, and I tried it too. It’s horrible.* (P149)

It can also be inferred that emotional vulnerability can trigger self-destructive thoughts and behaviors, as evidenced in the following account of a suicide attempt after the loss of a loved one:

[...] *I took all the medicine I had in my drawer* [...] *because I lost a very special person in my family, and I wanted to go with her.* (P81)

#### 2.5 Psychosomatic complaints arising from distress

Traumatic events, such as the loss of a family member or experiencing violence, can generate significant psychosomatic symptoms. One adolescent, for instance, associated abdominal pain with arguments with his mother:

[Stomach ache] *was an argument. I went to argue with my mother.* (P118)

Another described an anxiety crisis stemming from negative social interactions:

[...] *my vision blurred, my mother took me to the hospital, and the doctor said I was just very nervous* [...] *generally, it happens because of other people. Like, someone comes and says something that really, let’s say, weighs heavily on me or makes me very nervous, and a panic attack comes. So, it’s not something I can control.* (P154)

It is also noted that isolation and social exclusion contribute to symptoms such as fatigue and panic attacks:

*Last time, it was because I was feeling very lonely. I was sitting in the middle of the group of adolescents. I started to feel very alone. And I started to have a breakdown because my friends were back there and I couldn’t sit with them. I felt surrounded by people. I saw a lot of people giving me dirty looks, I didn’t know why* [...] *I started to panic.* (P156)

#### 2.6 Mental distress related to family health

Research has shown that family illnesses and accidents cause worry and fear, as reported by an adolescent:

*My mother had an accident in the morning and my grandmother had an accident in the afternoon of the same day, but my mother only hurt her arm and foot. My grandmother had an accident, lost consciousness, and is in the hospital. She’s 60 years old, so she has high blood pressure. Oh my God, I’m waiting for her to recover. But, my God, what bad luck. Please help me.* (P104)

Another person expressed anxiety upon seeing the Mobile Emergency Care Service, fearing for their mother:

*I worry a lot about things* [...] *yesterday, my mother wasn’t feeling well with her back* [...] *my mother has kidney problems* [...] *the first time, she called the ambulance* [...] *my mother said that if she doesn’t get better, she still wants to go to the ambulance* [...] *then I was on the street last night* [...] *then an ambulance passed by, you know, suddenly, I felt desperate, scared, a little nervous,* [...] *I went to see if there had been any messages, nothing. So, the ambulance was coming this way, I thought the ambulance was going to turn around* [...] *and the ambulance went straight past* [...] *if it had turned around, my God.* (P136)

Another adolescent reinforced this point by expressing concern about his hospitalized aunt:

[...] *I get worried about things. Especially about my aunt who’s getting sick.* [...] *she was hospitalized for a while, with the device stuck in her chest and nose. That made me worried. My God, will she be okay? I don’t know. I’m thinking far ahead, will she die? I’m so scared.* (P123)

They also expressed fear of developing mental health problems due to their family history:

*I’m afraid of developing, you know what? Mental health problems, because my family has a strong history of them. I’m afraid of having something wrong with my head, of having a breakdown.* (P140)

And another adolescent reported feeling guilty about his smoking mother’s illness:

[...] *my mother has a spot on her lung, that’s why I don’t smoke, because she smokes. And she has a spot on her lung; it could cause lung cancer. I kept blaming myself because I wasn’t there for her, telling her to stop smoking.* (P52)

## DISCUSSION

This study, based on Solar and Irwin’s SDH model^(13)^, showed that adolescents experience mental distress related to the search for economic stability and uncertainty about the future. The constant pressure for academic performance, college admission, job acquisition, and family responsibilities contributes to recurring thoughts and feelings of helplessness in the face of the future. Stressful situations experienced early in life favor the release of hormones such as cortisol, potentiating symptoms of anxiety^(16)^, which manifest as apprehension, fear, and discomfort^(17)^. Studies indicate that thinking about the future is strongly linked to the emotional state of young people, exerting more influence on their mood than the other way around^(18)^. Furthermore, the way adolescents perceive the future - including feelings of hopelessness, optimism, or a sense of control - is directly linked to the presence of anxiety and depressive symptoms^(19)^.

Interpersonal relationships have proven to be a determining factor in the recurring thought patterns among adolescents. Family conflicts, insecurity in friendships, and pressures related to academic performance were identified as factors that negatively affect self-esteem and emotional resilience. Although social support can act as a protective factor, pressure from parents and friends can also hinder reflection on the future^(18)^. Adolescents reported greater trust in friends than in family members when seeking emotional support. Even so, many young people developed adaptive strategies based on past experiences of psychological distress, using these experiences to challenge negative thoughts and strengthen their resilience. This capacity is associated with a reduction in depressive symptoms and stable clinical remission in adulthood^(18)^.

Furthermore, academic performance was shown to be directly impacted by symptoms of anxiety and depression, demonstrating that adolescents with a higher prevalence of these conditions tend to have lower academic performance. According to Horn, Silva and Patias^(20)^, these symptoms compromise quality of care, hindering both learning and retention of content, and are associated with self-demand and feelings of inadequacy. Such evidence reinforces the relationship between academic performance and physical symptoms of anxiety identified in the present study.

The absence of effective public policies and insufficient social protection were identified as structural factors that exacerbate adolescents’ mental distress. Reports revealed feelings of insecurity and frequent exposure to violence in school and urban environments, generating constant fear and contributing to symptoms of anxiety and post-traumatic stress. The literature supports these findings, indicating that exposure to violence negatively impacts young people’s neurodevelopment and psychological well-being^(21,22)^. According to Riehm *et al*.^(23)^, adolescents reported extreme concern about episodes of violence in schools, associating this fear with an increased risk of anxiety disorders.

Furthermore, school violence has been linked to low motivation to learn, reduced academic engagement, and a greater propensity for developing chronic illnesses and mental disorders in adulthood^(24)^. Such evidence reinforces the need for structural actions, such as mental health programs, inclusion initiatives, anti-bullying policies, and specific legislation, to promote safe school settings.

Similarly, anger and aggressive behavior among adolescents are psychosocial responses often linked to adverse experiences, such as conflicting family dynamics, social exclusion, and lack of emotional support. Studies indicate that these behaviors are associated with difficulties in emotional regulation, peer influence, and deficits in communication skills^(25,26)^. Interventions aimed at anger management have proven effective in reducing aggressive behaviors and promoting emotional adjustment^(25)^. In this context, the family emerges as a determining factor, capable of mitigating or intensifying the psychological distress of adolescents^(27)^. Dysfunctional environments, ongoing stress, and a lack of social support make it difficult to develop healthy coping strategies.

Furthermore, bullying, as a social phenomenon, profoundly impacts adolescents’ mental health, causing feelings of helplessness, anxiety, and exclusion. Prolonged experiences of school violence are linked to disorders such as depression, insomnia, and post-traumatic stress^(28)^. Unstructured school settings, coupled with a lack of support, perpetuate this distress. Support from health and education professionals, as well as strategies such as assertive therapy, have proven effective in preventing and mitigating the harm caused by bullying^(29)^. Nurses’, psychologists’, and professors’ work is fundamental, both in providing emotional support to victims and in promoting more respectful and inclusive relationships in school settings.

Similarly, dissatisfaction with self-image, intensified by rigid aesthetic standards and exposure to social media, is a significant factor in adolescents’ psychological distress, especially girls. This social pressure can lead to low self-esteem, a desire for body modification, and feelings of inadequacy^(30)^. Judgment within school settings also exacerbates this negative perception. On the other hand, policies such as restricting cell phone use in schools and strengthening family support have the potential to mitigate these impacts. Redefining self-image involves building affective bonds and promoting self-esteem.

It is also noted that feelings such as sadness, guilt, loneliness, and rejection are directly associated with non-suicidal self-harm among adolescents, and are aggravated by excessive punishment, family conflicts, and social exclusion. Studies show that anxiety and depression are factors strongly correlated with suicidal ideation^(31)^. Witnessing suicide among peers also profoundly affects the emotional well-being of young people, increasing the risk of mental health disorders^(32)^. The absence of consistent support networks and adequate coping strategies highlights the importance of supportive environments, family emotional support, and access to mental healthcare services as forms of prevention^(33)^.

Adolescents reported psychosomatic symptoms such as abdominal pain and fatigue associated with episodes of anxiety and sadness. These signs may indicate the development of mental disorders in adulthood, reinforcing the importance of early intervention^(34)^. Early recognition and attentive listening by healthcare professionals are fundamental for the proper reception and referral of adolescents.

Family members’ illness, especially due to mental illness, causes profound distress in adolescents, leading to symptoms of anxiety and sadness. Stigma by association, i.e., the prejudice faced by relatives of people with mental disorders, intensifies these young people’s distress, generating shame, fear, and concealment of the family’s condition^(35)^. Resilience, while important, is not sufficient on its own. Family, social, and professional support is essential to protect these adolescents’ mental health^(36)^. Distress is intensified by structural factors, such as financial difficulties, family changes, and barriers to accessing care, pointing to the need for integrated public policies that are sensitive to the social realities of young people.

### Study limitations

This study has limitations, as the interviews did not originate from specific questions about SDH involved in mental distress; however, the variety of records allowed for adequate responses to the proposed objective. Furthermore, the findings do not allow for generalizations, as they refer to data from a single Brazilian municipality.

### Contributions to nursing, health, or public policy

Analyzing SDH related to mental distress in adolescence can help child and adolescent mental healthcare professionals and primary care providers to expand care, strengthening psychosocial attention and acting both in the prevention and coping with distress. Furthermore, it contributes to strengthening nurses’ role as a leader in health promotion through strategies focused on social determinants.

## FINAL CONSIDERATIONS

The study highlights the strong influence of SDH on adolescent mental health, emphasizing the role of school settings. By using the Solar and Irwin model, it was identified that structural determinants, such as economic instability, family and educational context, directly affect emotional well-being, generating concerns about the future and increasing social pressure. On the other hand, positive interpersonal relationships, especially with family and support networks, act as protective factors. Among the intermediate determinants, aggressive behaviors, difficulties in emotional regulation, and negative self-image exacerbate psychological distress. School settings can be both a space for development and a source of stress, especially in contexts of bullying, pressure for performance, and exclusion. Exposure to violence also increases the risk of disorders such as anxiety and depression. The study reinforces the importance of public policies and intersectoral actions that promote mental health, strengthening support networks and ensuring a welcoming and protective school and family environment.

## Data Availability

The research data are available only upon request.
