# Supplementary material for: Social determinants of health that permeate adolescent students’ mental distress
Source: Rev Bras Enferm. 2026 Jul 17;79:e20250281. doi: 10.1590/0034-7167-2025-0281 (PMC13379242; doi:10.1590/0034-7167-2025-0281)
Supplement: Translation

# Translation: Determinantes sociais da saúde que permeiam o sofrimento mental de adolescentes escolares

**DOI:** 10.1590/0034-7167-2025-0281pt

**Authors:** Luiza Maria Rabelo Silva, Sabrina Silva Martins, Larissa de Almeida Rezio, Pâmela Roberta Oliveira, Marina Nolli Bittencourt

**Affiliations:** I Universidade Federal de Mato Grosso. Cuiabá, Mato Grosso, Brasil; II Universidade Federal de Mato Grosso. Barra do Garças, Mato Grosso, Brasil

**Keywords:** Determinantes Sociais da Saúde, Adolescente, Saúde Mental, Promoção da Saúde no Ambiente Escolar, Sofrimento Emocional.

## Abstract

**Objetivos:**

analisar os determinantes sociais da saúde envolvidos no sofrimento mental de adolescentes escolares no município de Cuiabá.

**Métodos:**

estudo qualitativo, vinculado a projeto matricial, fundamentado no modelo teórico de determinantes sociais da saúde de Solar e Irwin. Os dados foram provenientes de pesquisa matriz, sendo analisadas 98 entrevistas com adolescentes de 12 a 18 anos. As entrevistas foram guiadas pelo instrumento de autoavaliação de aspectos emocionais e competências sociais: *Youth Self-Report*. A análise de dados qualitativos se deu por meio da análise temática proposta por Braun e Clarke.

**Resultados:**

à luz do modelo teórico adotado, da categoria determinantes estruturais de saúde, emergiram três subcategorias, e da categoria determinantes intermediários de saúde, seis subcategorias.

**Considerações Finais:**

ao utilizar o modelo de Solar e Irwin, foi possível identificar que os determinantes sociais da saúde desempenham importante papel na manifestação de sintomas de saúde mental entre adolescentes.

## INTRODUÇÃO

A abordagem dos determinantes sociais da saúde (DSS) amplia a compreensão do processo de saúde-doença ao interligar saberes de múltiplas áreas, como políticas públicas, aspectos sociais, econômicos e culturais. Essa perspectiva considera as condições de vida ao longo de todo o ciclo vital, moldando a incidência de doenças e os riscos à saúde coletiva^(1)^. Nesse sentido, a Organização Mundial da Saúde (OMS) criou uma comissão dedicada a investigar os efeitos dos DSS nos estágios iniciais da vida, destacando fatores que afetam o desenvolvimento infantil em contextos familiares, escolares, comunitários e sociopolíticos^(2)^.

Pesquisas demonstram que rupturas em contextos associados aos DSS podem desestabilizar a realidade vivenciada pelos indivíduos, elevando a vulnerabilidade a infecções, transtornos mentais, distúrbios alimentares, uso de drogas, automutilação, iniciação sexual prematura e gestação na adolescência^(3)^.

Na última década, o crescimento de transtornos psiquiátricos reposicionou o adoecimento mental como questão social e de saúde pública global. Além de direito fundamental, a saúde mental é um pilar para o progresso individual e socioeconômico das comunidades^(4)^.

Dessa forma, analisar as repercussões dos DSS na saúde mental em diferentes fases da vida é primordial, pois a suscetibilidade a esses fatores varia conforme a trajetória individual^(5)^. Ademais, destaca-se que eventos ocorridos em períodos críticos do desenvolvimento, como a primeira infância, possuem efeitos mais graves no desencadeamento de problemas mentais^(6)^.

Nesse sentido, Prokosck *et al*.^(7)^ identificaram que situações estressantes e traumas na infância prejudicaram a saúde mental de jovens adultos, desencadeando ansiedade e condutas disruptivas. Durante a pandemia, adolescentes no Quênia e na Nigéria^(8)^ enfrentaram agravamento de condições sociais e novos desafios, como fome, suspensão escolar e isolamento social, o que intensificou quadros de ansiedade, gestações e uniões precoces, além de episódios de abusos físicos, sexuais e emocionais^(8)^. De forma similar, no Oiapoque, extremo norte brasileiro, evidenciou-se que determinantes como abandono, violência, *bullying*, fome e abuso infantil estiveram diretamente associados ao sofrimento psíquico em crianças^(3)^.

Tais evidências reforçam a necessidade de políticas públicas que promovam a saúde mental e ofereçam apoio psicossocial a crianças e adolescentes, por meio da articulação entre educação, saúde e assistência social. Como resposta a essa demanda, a Lei nº 14.819, sancionada em janeiro de 2024, instituiu a Política Nacional de Atenção Psicossocial nas Comunidades Escolares, promovendo ações integradas entre escolas, o Programa Saúde na Escola, o Sistema Único de Assistência Social e as redes de atenção psicossocial^(9)^.

A sinergia entre setores e a priorização do ambiente escolar são estratégias-chave, já que a escola é um espaço central para o desenvolvimento cognitivo e emocional, além de permitir o rastreamento de riscos e fatores protetivos.

Diante das lacunas e desafios destacados pela literatura, como a formação profissional inadequada, a falta de integração entre redes de apoio e a carência de diretrizes políticas claras, torna-se essencial compreender os DSS que influenciam a saúde mental dos adolescentes, a fim de embasar intervenções mais eficientes. Nesse sentido, a pesquisa busca responder à seguinte questão: de que forma os DSS mental se expressam entre adolescentes estudantes de Cuiabá à luz do modelo de DSS desenvolvido por Solar e Irwin?

## OBJETIVOS

Analisar os DSS envolvidos no sofrimento mental de adolescentes escolares no município de Cuiabá, Mato Grosso, Brasil.

## MÉTODOS

### Aspectos éticos

O presente estudo integra um projeto matricial previamente autorizado pela Secretaria Estadual de Educação e aprovado pelo Comitê de Ética em Pesquisa da Universidade Federal do Mato Grosso. Por utilizar dados secundários, dispensou nova autorização ética, mantendo os princípios de confidencialidade, anonimato e não maleficência, conforme a Resolução nº 466/2012. A pesquisa respeitou o direito à informação, privacidade, participação voluntária e retirada do consentimento, sem prejuízos e sem riscos à saúde dos participantes.

### Referencial teórico-metodológico

Os DSS refletem uma visão ampliada do processo de saúde-doença^(10)^, e, segundo Buss e Pellegrini Filho^(11)^, referem-se às condições nas quais as pessoas “nascem, crescem, vivem, trabalham e envelhecem”, influenciadas por fatores sociais, econômicos, comportamentais, culturais, étnicos/raciais e psicológicos que afetam diretamente a saúde e qualidade de vida.

Atualmente, a OMS adota o modelo de Solar e Irwin, apresentado na Conferência Mundial sobre Determinantes Sociais da Saúde em 2011^(12)^, o qual distingue determinantes estruturais e intermediários. Enquanto os estruturais incluem fatores como políticas públicas, sistemas de governança, mercado de trabalho, educação e valores sociais que moldam a estratificação socioeconômica^(13)^, os intermediários envolvem condições materiais de vida e trabalho, fatores psicossociais e comportamentais, e o sistema de saúde. Ressalta-se que este último é considerado um determinante intermediário, devido às barreiras de acesso, e que a coesão e o capital social atravessam as dimensões do modelo^(13)^.

Por fim, a Comissão Nacional sobre Determinantes Sociais da Saúde^(12)^ destaca os determinantes estruturais como centrais para compreender as iniquidades em saúde, resultantes de assimetrias de poder e do contexto socioeconômico e político. No caso do sofrimento mental entre adolescentes escolares, esse modelo evidencia como pobreza, violência, instabilidade familiar e ambiente escolar impactam negativamente a saúde mental. Redes de apoio e acesso a serviços de saúde mental, por outro lado, podem reduzir esses efeitos, ressaltando a importância de ações intersetoriais para promoção de um ambiente escolar acolhedor e equitativo.

### Tipo de estudo

Estudo qualitativo, de cunho descritivo e exploratório, tendo como referencial teórico o modelo de DSS de Solar e Irwin^(13)^.

### Procedimentos metodológicos

#### Cenário de estudo

Ocorreu em sete escolas regulares de ensino médio da rede estadual, localizadas em áreas urbanas da capital de Mato Grosso, das quais três funcionavam em tempo integral. Essas escolas foram selecionadas por conveniência.

#### Fonte de dados

Os dados foram captados de banco de dados com 222 entrevistas com adolescentes entre 12 e 18 anos, faixa etária definida com base no Estatuto da Criança e do Adolescente^(14)^, e nos fundamentos teóricos do *Youth Self-Report* (YSR)^(15)^, que foi o instrumento que guiou as entrevistas. Foram incluídas nesta pesquisa as entrevistas de adolescentes que apresentaram respostas indicativas de sofrimento mental segundo o YSR e que as relacionava com vivências que permitiram correlacionar os sintomas aos DSS. Foram excluídas entrevistas incompletas e com áudios de baixa qualidade.

#### Coleta e organização de dados

A coleta de dados referente às entrevistas nas escolas ocorreu em duas etapas. A primeira ocorreu entre março de 2021 e dezembro de 2022 em sete escolas estaduais da capital de Mato Grosso, com equipe composta por acadêmicas de enfermagem, uma docente e um doutorando. Após seleção das escolas e autorização dos diretores, os pesquisadores apresentaram o estudo aos professores e alunos, distribuindo o Termo de Consentimento Livre e Esclarecido (TCLE) para os responsáveis. Alunos com TCLE e Termo de Assentimento Livre e Esclarecido assinados participaram da coleta.

As entrevistas e a aplicação dos instrumentos ocorreram em salas reservadas, com duração média de 45 minutos. Os dados foram coletados digitalmente via *Google Forms^®^*, utilizando o YSR. As entrevistas foram gravadas, e os relatos relevantes foram aprofundados com a pergunta aberta “Fale-me mais sobre isso”.

A segunda etapa começou após a transcrição dos áudios. Das 222 entrevistas realizadas, 98 adolescentes apresentaram sintomas psicopatológicos, e 32 entrevistas foram selecionadas para análise, por mostrarem relações entre sofrimento mental e DSS, conforme o modelo de Solar e Irwin^(13)^.

#### Análise de dados

Os dados foram examinados por meio da análise temática de Braun e Clarke^(16)^, na qual, nas fases um a quatro, utilizou-se como base teórica o modelo de DSS de Solar e Irwin^(13)^. A aplicação dessa metodologia se desdobrou em seis fases, e a análise revelou duas categorias principais. A categoria 1, referente aos determinantes estruturais das desigualdades em saúde, abrange três subcategorias: Pensamentos recorrentes de preocupação com o futuro; Influência dos relacionamentos interpessoais na recorrência de pensamentos; e Baixa proteção social como desencadeadora de sofrimento mental. A categoria 2, correspondente aos determinantes intermediários da saúde, inclui seis subcategorias: Comportamento agressivo e raiva como consequência de experiências vividas; Vivência do *bullying* e repercussões no sofrimento mental; Impacto da autoimagem na saúde mental; Fatores psicossociais e autolesão não suicida; Queixas psicossomáticas decorrentes do sofrimento; e Sofrimento mental relacionado à saúde da família.

## RESULTADOS

### 1 - Determinantes estruturais das desigualdades em saúde

Essa categoria abrange posição socioeconômica, políticas públicas, cultura e valores.

#### 1.1 Pensamentos recorrentes de preocupação com o futuro

Os adolescentes demonstraram angústia em relação ao futuro, revelando preocupações com emprego, estudos e responsabilidades familiares:

*Preocupação em arranjar emprego e ajudar os pais, fazer um curso e sei lá.* (P99)

*Preocupação com o futuro* [...] *com faculdade, com estudo, com trabalho.* (P103)

O P6 revelou preocupação excessiva com o papel de cuidador na família, indicando responsabilidade precoce, mesmo em detrimento de suas próprias necessidades:

[...] *como que eu vou viver com minha família, sendo que eu sou meio que o cuidador da casa e eu tenho que ficar lá pra ajudar eles* [...] *então, eu só quero dar orgulho pra eles* (P6).

De forma semelhante, o P15 revelou uma cobrança constante por desempenho em áreas como estudos, trabalho e cuidados pessoais, enquanto P118 demonstrou uma autopercepção marcada por exigência quanto ao desempenho escolar:

[...] *eu fico o tempo todo pensando assim, ah, eu preciso fazer dar certo. Tipo, esse ano tem o Enem; eu quero passar* [...] *eu preciso me dedicar, eu preciso dar o meu melhor em tal coisa. Ou então, se eu tô no trabalho, eu fico, tipo assim: “Aguenta firme, não surta”* [...] *na academia, eu fico, tipo assim: “Ah, vai, você consegue”. É sempre assim*. (P15)

[...] *penso muito sobre a escola, acho que eu penso demais sobre as minhas notas. Acho que eu me cobro muito ainda sobre isso.* (P118)

Além da autocobrança, os relatos de P15 e P118 indicaram tentativa de repressão emocional, como nas expressões “aguenta firme” e “não surta”.

#### 1.2 Influência dos relacionamentos interpessoais na recorrência de pensamentos e autopercepção

As relações interpessoais influenciam os pensamentos recorrentes dos adolescentes, sendo os conflitos familiares, a insegurança nas amizades e o desempenho acadêmico identificados como os principais fatores desencadeadores, conforme a seguinte resposta:

*Preocupações sobre minha família, sobre minhas amigas*. (P139)

A insegurança nas interações apareceu em respostas como:

*Se eu mandar alguma mensagem para alguma pessoa, e a pessoa não me responder ou me responder de um jeito diferente do que eu imaginava, eu penso se eu mandei a mensagem certa ou se eu fiz a escolha certa.* (P100)

*Fico comparando muito, e tem vez que eu sinto que as pessoas me deixam de lado* [...] *tenho uma amiga que conto tudo para ela. Nisso, a gente tem outra amiga e ela tipo meio que me deixa de lado. Eu fico muito mal com isso.* (P125)

Já as comparações sociais surgiram como fator significativo, como mostra o relato de P125, revelando o sentimento de baixa autoestima, insegurança ou de não se sentir querida. Além disso, o desempenho acadêmico também é uma preocupação constante, associado a sintomas físicos de ansiedade e dificuldades cognitivas, conforme relatado por P142:

*Tipo quando eu tiro nota baixa. Aí eu fico preocupado. Quer dizer, antes de chegar na prova, eu fico preocupado.* [...] *dá ansiedade, eu fico suando*. (P142)

Ademais, a autorreflexão constante sobre erros passados surgiu em várias falas, como a do P122 e do P153:

*Às vezes, eu faço alguma coisa errada, daí eu fico tipo, sei lá, uma semana pensando naquilo*. (P122)

*Tipo, se eu errar alguma coisa, eu fico pensando*. (P153)

Destacou-se ainda que a necessidade de validação externa impacta a autoimagem e a autoestima, como evidenciado por P149:

[...] *se a pessoa me elogiar, eu vou ficar remoendo aquilo até eu achar o mesmo. E se a pessoa falar que eu sou boa em tal, eu vou ficar remoendo aquilo até eu ficar convencida e não me achar mais boa*. (P149)

#### 1.3 Baixa proteção social como desencadeadora de sofrimento mental

A escola apareceu como espaço de insegurança, gerando medo e estresse:

[...] *me preocupo de uma pessoa vir e fazer um massacre aqui na escola. Porque aconteceu esse dia na IFMT. Um menino trancou todos os alunos numa sala* [...] *os alunos da mesma sala e o professor, e tentou esfaquear eles.* (P101)

*Eu tenho* [medo] *porque alguém já foi esfaqueado aqui já. Não sei se foi com uma faca ou com uma caneta, tanto que foi bem ali naquela sala, acho que foi na sala da escola. A sala doze, essa daqui assim, e, mano, tá lotado de sangue. Me mostraram os vídeos, o vídeo, tanto que a menina já até foi presa.* (P157)

A sensação de insegurança também se estende a outros espaços sociais, como as ruas, onde os adolescentes vivenciaram situações de assédio e abuso, sendo o medo da violência de gênero evidenciado no relato da participante P139:

*Tenho medo de alguém me abusar na rua. É, eu tenho muito medo de eu estar andando, aí alguém vai lá, me pega. E também de assédio. Tenho muito medo* [...] *um dia, eu tava vindo pra escola com a minha mãe* [...] *um carro preto passou do meu lado e minha mãe tava mais na frente, quando eu tô devagar* [...] *ele falou: “Aí, gatinha”, eu fiquei com medo, eu comecei a chorar.* (P139)

Além disso, a má condição das vias públicas também contribui para a insegurança:

*Eu tenho medo de buracos. Bueiro mais específico. Que eu caí num bueiro. Eu tava olhando pra lua*. [...] *era um buraco que envolvia minha perna. Levei 54 pontos.* (P30)

### 2 - Determinantes intermediários da saúde

Esta categoria aborda fatores comportamentais, psicossociais e circunstâncias materiais.

#### 2.1 Comportamento agressivo e raiva como consequência de experiências vividas

Os adolescentes relataram que interações negativas geraram irritação, raiva e pensamentos agressivos associados a dinâmicas familiares conflituosas, rejeição e falta de suporte emocional.

Um adolescente exemplificou a frustração diante de tratamento injusto:

[...] *por algum motivo, eu fico bastante irritada quando eles me tratam ruim e com ignorância.* (P140)

Outro adolescente expressou medo da própria raiva e dos pensamentos agressivos que ela pode gerar:

[...] *eu tenho medo de pegar uma faca e esfaquear alguém. Eu tenho medo de empurrar alguém na frente de um carro. Eu tenho medo de chegar no quarto da minha mãe e bater nela de raiva. Aí vai ser um surto, porque eu tenho muita raiva também de levantar naquela igreja e bater na cara de uma criança, porque criança que faz muito escândalo pra mim merece levar umas boas surras, porque eu tenho um certo trauma por causa disso.* (P149)

#### 2.2 Vivência do *bullying* e repercussões no sofrimento mental

As falas apontaram que o *bullying* escolar agrava sentimentos negativos, mas o apoio familiar ajudou a ressignificar essas experiências.

*Me viam, olharam no olho e faziam esse som de vômito. Que ódio.* [...] *fui muito motivo de bullying na minha antiga escola. Aí minha madrasta virou e falou que não era pra mim ligar, porque eu era uma menina muito especial e que a opinião deles não valia. Aí eu ficava repetindo isso na minha cabeça* [...] *tem muita gente que não gosta de mim, tipo, 95% da escola.* (P52)

O *bullying* também gera insegurança corporal, como relatado por P154:

[...] *as pessoas ficam me zoando e eu não gosto disso*. [...] *eu sou muito insegura com o meu corpo*. (P154)

Um relato ainda apontou que o *bullying* pode, em alguns casos, levar a pensamentos suicidas:

*Eu já tentei* [...] *teve uma coisa que já me magoou muito ao ponto de eu querer realmente: foi o bullying. Eu sofri muito bullying quando eu era pequena* [...] *eu sofri agressão na escola, das crianças, porque eu era gordinha*. (P7)

#### 2.3 Impacto da autoimagem na saúde mental

Evidenciou-se também que a insatisfação com a aparência gera sofrimento e baixa autoestima, como demonstra o seguinte relato:

[...] *eu acho que eu sou muito feia*. [...] *parece que tem roupa que não fica adequada em mim. Tipo, eu acho que eu sou muito gorda* [...] *então, parece que quando eu fico de calça eu fico feia, porque tem outras meninas que ficam bonitas*. (P106)

Além disso, gera o desejo de ter um corpo diferente, conforme relatado:

*Queria ser muito magra* [...] *queria pesar 48 quilos, tô tentando chegar nessa meta*. [...] *queria ter, mas eu não tenho anorexia nervosa*. (P24)

#### 2.4 Fatores psicossociais e autolesão não suicida

As entrevistas revelaram que rejeição, conflitos familiares e influência de amizades estão associados à autolesão. Na fala a seguir, a sensação de rejeição e julgamento pelos outros se manifestou como um fator no sofrimento psíquico:

[...] *porque eu sinto que as pessoas não gostam de mim, que todo mundo tá me julgando, que todo mundo olha com canto de olho pra mim. Porque, basicamente, eles não entendem o que eu tô sentindo.* (P154)

Além disso, a exclusão social por parte da família, como fator desencadeante de autolesão, restou evidenciada na seguinte resposta:

[...] *e o que mais faz eu ter esses pensamentos e fazer essas coisas* (autolesão) *são meus pais, porque às vezes eu sinto que meu irmão é, tipo, preferência pra eles e eles preferem mais meu irmão do que eu, entendeu?* [...] *e aí eu me sinto muito triste. Me sinto excluída* (P132)

Ainda pelos relatos, verifica-se que conflitos familiares são gatilhos para autolesão não suicida:

[...] *aconteceu assim: eu estava em casa e minha mãe veio falar comigo. Eu não sei o que aconteceu, que eu fiquei com raiva dela, e eu falei um negócio que deixou ela chorando. Eu fiquei chorando a noite inteira. Eu me arrependi bastante, tanto que até eu me cortei num dia desse por causa disso que aconteceu.* (P157)

[...] *eu quero muito, muito, muito ingerir doses altíssimas de remédios só pra viajar, porque minha realidade com a minha mãe é muito difícil.* (P24)

Ademais, há relatos das influências das amizades nesse comportamento:

[...] *às vezes, eu brigava com meus pais, e esses amigos virtuais que eu tenho, tinha um amigo* [...] *a gente se conheceu no Instagram^®^ e a gente conversava bastante, só que* [...] *a gente brigou e só fiquei muito triste e eu me cortei.* (P132)

*Eu já me cortei, mas foi só por causa das pessoas. Mas porque eu vi uma amiga fazendo, ela falou que era bom, eu fui tentando fazer. É horrível.* (P149)

Infere-se ainda que a vulnerabilidade emocional pode desencadear pensamentos e comportamentos autodestrutivos, como evidenciado no relato que se segue de tentativa de suicídio após a perda de um ente querido:

[...] *eu tomei todos os remédios que tinha na minha gaveta* [...] *porque eu perdi uma pessoa muito especial na minha família, e aí eu queria ir junto com ela.* (P81)

#### 2.5 Queixas psicossomáticas decorrentes do sofrimento

Eventos traumáticos, como a perda de um familiar ou a vivência de violência, podem gerar sintomas psicossomáticos importantes. Um adolescente, por exemplo, associou dores abdominais a discussões com a mãe:

[Dor de barriga] *foi discussão. Fui discutir com a minha mãe.* (P118)

Outro descreveu crise de ansiedade por interações sociais negativas:

[...] *minha vista embaçou, minha mãe me levou no hospital e o médico falou que eu só estava muito nervosa* [...] *geralmente, ocorre por conta das outras pessoas. Tipo, alguém vem e fala algo que realmente, digamos, que pesa muito ou que me deixa muito nervosa e a crise de pânico vem. Então, não é algo que eu consigo controlar.* (P154)

Nota-se também que o isolamento e a exclusão social contribuem para sintomas como fadiga e crises de pânico:

*Na última, foi porque eu estava me sentindo muito sozinha. Eu estava sentada no meio do conjunto dos adolescentes. Eu comecei a me sentir muito sozinha. E eu comecei a entrar em crise, porque os meus amigos estavam lá atrás e eu não conseguia sentar junto deles. Eu estava me sentindo rodeada de gente. Eu estava vendo um monte de gente me olhando com cara feia, eu não sabia o motivo* [...] *comecei a entrar em pânico.* (P156)

#### 2.6 Sofrimento mental relacionado à saúde da família

As pesquisas demonstraram que doenças e acidentes familiares provocam preocupação e medo, como relatou adolescente:

*A minha mãe se acidentou de manhã e minha avó se acidentou à tarde, no mesmo dia, só que minha mãe só machucou o braço e o pé. A minha avó bateu, ficou inconsciente e tá no hospital. Ela tem 60 anos, então tá com a pressão alta. Nossa, eu tô esperando pra ela se recuperar. Mas, meu Deus, que azar. Vem me ajudar.* (P104)

Outro ainda expressou ansiedade ao ver o Serviço de Atendimento Móvel de Urgência, temendo pela mãe:

*Eu me preocupo muito com as coisas* [...] *ontem, minha mãe não estava bem com as costas* [...] *minha mãe tem problema de rim* [...] *na primeira vez, chamou o SAMU* [...] *minha mãe pegou e falou que, se ela não melhorar, ela ainda quer ir para o SAMU* [...] *aí eu estava ontem na rua ontem à noite* [...] *aí passou um SAMU, né, de repente, deu um desespero, um medo, um trem,* [...] *fui ver se tinha vindo alguma mensagem, nada. Então, o SAMU vindo lá para cá, eu achei que o SAMU ia virar* [...] *e o SAMU passou reto* [...] *se virasse, meu Deus.* (P136)

Outro adolescente reforçou essa questão ao expressar preocupação com a tia hospitalizada:

[...] *fico preocupada com as coisas. Com a minha tia que fica ruim*. [...] *ela ficou um tempo internada, com o trem enfiado no peito, no nariz. Aí eu fico preocupada. Meu Deus, será que ela vai ficar bem? Não sei. Eu tô pensando longe, será que ela vai morrer? Que medo*. (P123)

Ainda manifestaram medo de desenvolver problemas mentais devido ao histórico familiar:

*Eu tenho medo de ficar sabe o quê? Com problemas mentais, porque minha família tem muito histórico. Eu tenho medo de ter alguma coisa na cabeça, de surtar*. (P140)

E outro adolescente relatou culpa pela doença da mãe fumante:

[...] *minha mãe tá com mancha no pulmão, por isso que eu não fumo, porque ela fuma. E ela tá com uma mancha no pulmão, pode dar câncer no pulmão. Eu ficava me culpando, porque eu não estava lá perto dela, falando pra ela parar de fumar*. (P52)

## DISCUSSÃO

Este estudo, fundamentado no modelo de DSS de Solar e Irwin^(13)^, evidenciou que adolescentes vivenciam sofrimento mental relacionado à busca por estabilidade econômica e à incerteza quanto ao futuro. A constante pressão por desempenho escolar, ingresso na faculdade, conquista de um emprego e responsabilidades familiares contribuem para pensamentos recorrentes e sentimentos de impotência diante do futuro. Situações estressantes vivenciadas precocemente favorecem a liberação de hormônios como o cortisol, potencializando sintomas de ansiedade ^(16)^, que se manifestam como apreensão, medo e desconforto^(17)^. Estudos apontam que o pensamento sobre o futuro está fortemente relacionado ao estado emocional dos jovens, exercendo mais influência sobre o humor do que o contrário^(18)^. Além disso, a forma como os adolescentes percebem o futuro - incluindo sentimentos de desesperança, otimismo ou sensação de controle - está diretamente ligada à presença de sintomas ansiosos e depressivos^(19)^.

As relações interpessoais se mostraram determinantes no padrão de pensamento recorrente entre adolescentes. Conflitos familiares, insegurança nas amizades e pressões relacionadas ao desempenho acadêmico foram apontados como fatores que afetam negativamente a autoestima e a resiliência emocional. Embora o apoio social possa funcionar como fator protetivo, a pressão de pais e amigos também pode dificultar a reflexão sobre o futuro^(18)^. Os adolescentes relataram maior confiança em amigos do que em familiares ao buscar suporte emocional. Ainda assim, muitos jovens desenvolveram estratégias adaptativas com base em experiências passadas de sofrimento psíquico, utilizando essas vivências para desafiar pensamentos negativos e fortalecer sua resiliência. Tal capacidade está associada à redução dos sintomas depressivos e à remissão clínica estável na vida adulta^(18)^.

Ademais, o desempenho acadêmico se mostrou diretamente impactado pelos sintomas de ansiedade e depressão, evidenciando que adolescentes com maior presença dessas condições tendem a apresentar rendimento escolar inferior. De acordo com Horn, Silva e Patias^(20)^, esses sintomas comprometem a qualidade da atenção, dificultando tanto a aprendizagem quanto a retenção de conteúdos, e estão associados à autoexigência e a sentimentos de incapacidade. Tais evidências reforçam a relação entre o desempenho escolar e os sintomas físicos de ansiedade identificada no presente estudo.

A ausência de políticas públicas eficazes e a insuficiente proteção social foram identificadas como fatores estruturais que agravam o sofrimento mental dos adolescentes. Os relatos revelaram sentimentos de insegurança e exposição frequente à violência em ambientes escolares e urbanos, gerando medo constante e contribuindo para sintomas de ansiedade e estresse pós-traumático. A literatura corrobora esses achados, apontando que a exposição à violência impacta negativamente o neurodesenvolvimento e o bem-estar psicológico dos jovens^(21,22)^. Segundo Riehm *et al*.^(23)^, os adolescentes relataram preocupação extrema com episódios de violência em instituições escolares, associando esse medo ao aumento no risco de transtornos de ansiedade.

Além disso, a violência escolar tem sido vinculada à baixa motivação para aprender, menor engajamento acadêmico, e maior propensão ao desenvolvimento de doenças crônicas e transtornos mentais na vida adulta^(24)^. Tais evidências reforçam a necessidade de ações estruturais, como programas de saúde mental, iniciativas de inclusão, políticas *antibullying* e legislação específica, para promoção de ambientes escolares seguros.

Da mesma forma, a raiva e o comportamento agressivo entre adolescentes são respostas psicossociais frequentemente ligadas a experiências adversas, como dinâmicas familiares conflituosas, exclusão social e ausência de suporte emocional. Estudos apontam que esses comportamentos estão associados a dificuldades na regulação emocional, influências de pares e déficits em habilidades de comunicação^(25,26)^. Intervenções voltadas à gestão da raiva demonstram eficácia na redução de comportamentos agressivos e na promoção de adaptação emocional^(25)^. Nesse contexto, a família surge como um fator determinante, podendo mitigar ou intensificar o sofrimento psicológico dos adolescentes^(27)^. Ambientes disfuncionais, estresse contínuo e ausência de suporte social dificultam a construção de estratégias saudáveis de enfrentamento.

Outrossim, o *bullying*, enquanto fenômeno social, impacta profundamente a saúde mental dos adolescentes, provocando sentimentos de desamparo, ansiedade e exclusão. Prolongadas experiências de violência escolar estão relacionadas a transtornos como depressão, insônia e estresse pós-traumático^(28)^. Ambientes escolares desestruturados, somados à ausência de acolhimento, favorecem a perpetuação desse sofrimento. O suporte de profissionais de saúde e de educação, bem como estratégias como terapia assertiva, têm mostrado eficácia na prevenção e mitigação dos danos causados pelo *bullying* ^(29)^. A atuação de enfermeiros, psicólogos e professores é fundamental tanto no suporte emocional às vítimas quanto na promoção de relações mais respeitosas e inclusivas no ambiente escolar.

Do mesmo modo, a insatisfação com a autoimagem, intensificada por padrões estéticos rígidos e pela exposição às redes sociais, é um fator relevante no sofrimento psíquico dos adolescentes, especialmente entre as meninas. Essa pressão social pode gerar baixa autoestima, desejo de modificação corporal e sentimentos de inadequação^(30)^. O julgamento no ambiente escolar também agrava essa percepção negativa. Por outro lado, políticas como a restrição ao uso de celulares em escolas e o fortalecimento do apoio familiar têm potencial para atenuar tais impactos. A ressignificação da autoimagem passa pela construção de vínculos afetivos e promoção da autoestima.

Nota-se também que sentimentos como tristeza, culpa, solidão e rejeição estão diretamente associados à prática de autolesão não suicida entre adolescentes, sendo agravados por punições excessivas, conflitos familiares e exclusão social. Estudos demonstram que ansiedade e depressão são fatores fortemente correlacionados à ideação suicida^(31)^. A vivência do suicídio entre colegas também afeta profundamente o equilíbrio emocional dos jovens, ampliando o risco de transtornos mentais^(32)^. A ausência de redes de apoio consistentes e de estratégias adequadas de enfrentamento evidencia a importância de ambientes acolhedores, suporte emocional familiar e acesso a serviços de saúde mental como formas de prevenção^(33)^.

Os adolescentes relataram sintomas psicossomáticos como dores abdominais e fadiga associados a episódios de ansiedade e tristeza. Esses sinais podem indicar o desenvolvimento de transtornos mentais na vida adulta, reforçando a importância de intervenções precoces^(34)^. O reconhecimento precoce e a escuta qualificada por parte dos profissionais de saúde são fundamentais para o acolhimento e encaminhamento adequado dos adolescentes.

O adoecimento de familiares, especialmente por doenças mentais, causa profunda angústia nos adolescentes, levando a sintomas de ansiedade e tristeza. O estigma por associação, ou seja, o preconceito enfrentado pelos parentes de pessoas com transtornos mentais, intensifica o sofrimento desses jovens, gerando vergonha, medo e ocultação da condição familiar^(35)^. A resiliência, embora importante, não é suficiente isoladamente. O suporte familiar, social e profissional é essencial para proteger a saúde mental desses adolescentes^(36)^. O sofrimento é intensificado por fatores estruturais, como dificuldades financeiras, mudanças familiares e barreiras ao acesso ao cuidado, apontando para a necessidade de políticas públicas integradas e sensíveis às realidades sociais dos jovens.

### Limitações do estudo

Este estudo possui limitações, pois as entrevistas não foram originadas de perguntas específicas sobre os DSS envolvidos no sofrimento mental, entretanto a variedade de registros possibilitou respostas adequadas ao objetivo proposto. Além disso, os achados não permitem generalizações, pois referem-se a dados provenientes de um único município brasileiro.

### Contribuições para as áreas da enfermagem, saúde ou políticas públicas

A análise dos DSS relacionados ao sofrimento mental na adolescência pode auxiliar profissionais de saúde mental infanto-juvenil e de atenção primária a ampliar o cuidado, fortalecendo a atenção psicossocial e atuando tanto na prevenção quanto no enfrentamento do sofrimento. Além disso, contribui para fortalecer o papel do enfermeiro como líder na promoção da saúde, por meio de estratégias voltadas aos determinantes sociais.

## CONSIDERAÇÕES FINAIS

O estudo evidencia a forte influência dos DSS na saúde mental de adolescentes, com ênfase no papel do ambiente escolar. Utilizando o modelo de Solar e Irwin, identificou-se que determinantes estruturais, como instabilidade econômica, contexto familiar e educacional, afetam diretamente o bem-estar emocional, gerando preocupações com o futuro e aumentando a pressão social. Por outro lado, relações interpessoais positivas, especialmente com a família e redes de apoio, atuam como fatores de proteção. Entre os determinantes intermediários, comportamentos agressivos, dificuldades de regulação emocional e autoimagem negativa agravam o sofrimento psíquico. O ambiente escolar pode ser tanto um espaço de desenvolvimento quanto uma fonte de estresse, especialmente em contextos de *bullying*, pressão por desempenho e exclusão. A exposição à violência também eleva o risco de transtornos como ansiedade e depressão. O estudo reforça a importância de políticas públicas e ações intersetoriais que promovam a saúde mental, fortalecendo redes de apoio e garantindo um ambiente escolar e familiar acolhedor e protetor.

## Data Availability

Os dados de pesquisa só estão disponíveis mediante solicitação.
